# *Leishmania* infection-induced multinucleated giant cell formation *via* upregulation of ATP6V0D2 expression

**DOI:** 10.3389/fcimb.2022.953785

**Published:** 2022-09-23

**Authors:** Jing Hong, Chizu Sanjoba, Wataru Fujii, Junya Yamagishi, Yasuyuki Goto

**Affiliations:** ^1^ Laboratory of Molecular Immunology, Graduate School of Agricultural and Life Sciences, The University of Tokyo, Tokyo, Japan; ^2^ Laboratory of Applied Genetics, Graduate School of Agricultural and Life Sciences, The University of Tokyo, Tokyo, Japan; ^3^ International Collaboration Unit, International Institute for Zoonosis Control, Hokkaido University, Sapporo, Japan

**Keywords:** *Leishmania*, macrophage, multinucleated giant cell (MGC), ATP6V0D2, hemophagocytosis

## Abstract

Leishmaniasis is caused by infection with protozoan parasites of the genus *Leishmania*. In both clinical and experimental visceral leishmaniasis, macrophage multinucleation is observed in parasitized tissues. However, the feature and the mechanism of macrophage multinucleation remained unclear. Here, we report that infection of *Leishmania donovani*, a causative agent of visceral leishmaniasis, induces multinucleation of bone marrow-derived macrophages (BMDMs) *in vitro.* When these infection-induced multinucleated macrophages were compared with cytokine-induced multinucleated giant cells, the former had higher phagocytic activity on red blood cells but no apparent changes on phagocytosis of latex beads. BMDMs infected with *L. donovani* had increased expression of ATP6V0D2, one of the components of V-ATPase, which was also upregulated in the spleen of infected mice. Infection-induced ATP6V0D2 localized in a cytoplasmic compartment, which did not overlap with the mitochondria, endoplasmic reticulum, or lysosomes. When ATP6V0D2 expression was recombinantly induced in BMDMs, the formation of multinucleated macrophages was induced as seen in the infected macrophages. Taken together, *L. donovani* infection induces multinucleation of macrophages *via* ATP6V0D2 upregulation leading to a unique metamorphosis of the macrophages toward hemophagocytes.

## Introduction

Leishmaniasis is one of the most significantly neglected tropical diseases with more than one billion people living in the endemic areas. An estimated 30,000 new cases of visceral leishmaniasis and more than one million new cases of cutaneous leishmaniasis occur annually (WHO, 2022). It is caused by infection with protozoan parasites of the genus *Leishmania*, which maintain their life cycle through transmission between sandfly and a mammalian host. After the flagellated promastigotes invade the mammalian host, they transform into amastigotes which have non-exteriorized flagellum and typically live intracellularly in professional phagocytes including macrophages (Kaye and Scott, 2011). *Leishmania donovani* is one of the species to disseminate into internal organs such as the spleen, liver, and bone marrow and causes symptoms including hepatosplenomegaly and anemia (World Health Organization, 2010).

Multinucleated giant cells (MGCs) are cells that are formed by the fusion of monocyte/macrophage lineage (Helming and Gordon, 2008) and include osteoclasts responsible for bone resorption (Väänänen et al., 2000). MGC formation is also observed in non-skeletal tissues in many pathological processes including sarcoidosis and likely plays a number of roles in the pathogenesis of these diseases (Okamoto et al., 2003; Takano et al., 2004; Kawasaki and Purvin, 2009). According to morphological features and functional characteristics of MGCs, they are classified into several variants such as foreign-body giant cells (FBGCs), Langhans giant cells (LGCs), and Touton giant cells (Quinn and Schepetkin, 2009). These phenotypically different MGCs can be induced by stimulation with several cytokine combinations. For instance, FBGC formation is observed in BMDMs stimulated with interleukin (IL)-4 as well as granulocyte–macrophage colony-stimulating factor (GM-CSF) (Helming et al., 2009). By contrast, LGC formation is induced by interferon (IFN)-γ (Anderson, 2000).

Multinucleation of macrophages is reported in both clinical cutaneous leishmaniasis and visceral leishmaniasis. Binucleated cells and multinucleated giant cells were found in bone marrow aspiration samples of kala-azar patients infected with *L. donovani* (Sah et al., 2005; Daneshbod et al., 2010). Tissue sections of cutaneous leishmaniasis also showed granulomatous dermal infiltrate containing Langhans giant cells (Calvopina et al., 2006). MGCs are also found within granulomas in BALB/c mice experimentally infected with *Leishmania major* (Muraille et al., 2003). We have reported that MGCs are formed in the spleen of *L. donovani*-infected BALB/c mice (Morimoto et al., 2016). However, the factors that induce macrophage multinucleation and the functional roles of macrophage multinucleation during *Leishmania* infection remain unclear.

Several molecules are reported to have distinct functions in macrophage fusion for MGC formation (Pereira et al., 2018). Before starting cell fusion, macrophages are considered to shift to a pre-fusion state, which involves DAP12, TREM2, and KCNN4 (Helming et al., 2008; Kang et al., 2014). In the fusion process, attracting macrophages adjacent to each other seems to be an important element, and CCL2/CCR2 is involved in MGC formation (Kyriakides et al., 2004; Khan et al., 2016). To finalize cell–cell fusion, surface molecules including DC-STAMP act as fusion promoters (Yagi et al., 2005). It is also reported that ATP6V0D2 is indispensable in osteoclastogenesis as mice lacking the *Atp6v0d2* gene cannot form mature osteoclasts (Lee et al., 2006). ATP6V0D2 is a subunit of vacuole-ATPase which is a kind of proton pump maintaining the pH of the cytosol and intracellular acidic organelle (Vasanthakumar and Rubinstein, 2020), which is important for the activity of superoxide-generating enzymes.

We previously reported that the formation of MGCs was upregulated in the spleen of *L. donovani*-infected mice compared with that of uninfected mice, and those MGCs had increased activity to phagocytose erythrocytes (Morimoto et al., 2016), suggesting that the parasite-induced MGCs acquire the pathogenic characteristics of hemophagocytes distinct from osteoclasts. In this study, we used an *in-vitro* macrophage culture system to reproduce the MGC formation found *in vivo* and explore the mechanisms of the infection-induced macrophage multinucleation more precisely. In addition, significant upregulation of *Atp6v0d2* gene expression was found in the infected spleen, and we examined how *L. donovani* infection affects the expression of ATP6V0D2 by macrophages and how upregulation of the protein affects MGC formation *in vitro*.

## Materials and methods

### Ethics statement

All animal experiments were reviewed and approved by the Animal Experiment Committee at the University of Tokyo (Approval Nos. P17-076 and P20-063). The experiments were performed in accordance with the Regulations for Animal Care and Use of the University of Tokyo, which were based on the Law for the Humane Treatment and Management of Animals, Standards Relating to the Care and Management of Laboratory Animals and Relief of Pain (the Ministry of the Environment); Fundamental Guidelines for Proper Conduct of Animal Experiment and Related Activities in Academic Research Institutions (the Ministry of Education, Culture, Sports, Science and Technology); and the Guidelines for Proper Conduct of Animal Experiments (the Science Council of Japan). The collection of blood from mice was performed under anesthesia with isoflurane. At the end of the experiments, mice were euthanized by exsanguination under anesthesia with isoflurane followed by cervical dislocation.

### Mice, cells, and parasites

Female BALB/cA mice were purchased from Japan Clea, Tokyo, Japan. All mice were maintained under specific pathogen-free conditions. The mice were used for the experiments at the age of 6–8 weeks. Experimental infection of mice with *L. donovani* was performed as previously described (Morimoto et al., 2016).

Bone marrow (BM) cells were isolated from the femurs and tibias of BALB/cA mice. Bone marrow-derived macrophages (BMDMs) were generated by cultivating bone marrow cells in DMEM (Wako, Japan) supplemented with 10% heat-inactivated fetal bovine serum (HI-FBS, Thermo Scientific, USA), 100 U/ml of penicillin + 100 μg/ml of streptomycin (Wako) and 25 ng/ml of recombinant mouse macrophage colony-stimulated factor (M-CSF, PeproTech, USA) for 7 days at 37°C and 5% CO_2_. The medium was changed once with a fresh one on day 4.

*Leishmania donovani* promastigotes [MHOM/NP/03/D10, a gift from the National BioResource Project at Nagasaki University (Pandey et al., 2007)] were cultured in medium 199 (Invitrogen, USA) supplemented with 10% HI-FBS at 25°C. *Leishmania major* (MHOM/IL/80/Friedlin) were cultivated in medium 199 supplemented with 10% HI-FBS at 25°C. In some experiments, *Leishmania* promastigotes were stained with CFSE or CytoRed (Dojindo Laboratories, Japan). Promastigotes (1 × 10^7^) were incubated in 100 μl of DMEM medium containing 50 μg/ml of CFSE or CytoRed at room temperature for 30 min. The stained promastigotes were washed with DMEM three times and used for the *in-vitro* infection experiments.

### Histological analysis

Hematoxylin and eosin staining of mouse tissue sections was performed as described previously (Morimoto et al., 2016). The spleen and bone marrow of mice 24 weeks after *L. donovani* infection were fixed by 10% formalin and then embedded in paraffin. The tissues were sectioned at 4 μm sickness for hematoxylin and eosin staining. For immunohistochemical analysis, paraffin-embedded tissues were dewaxed and boiled in 10 mM of citrate acid buffer (pH 6.0) for 20 min. Endogenous peroxidase was inactivated with 0.3% H_2_O_2_ in methanol for 30 min. After blocking with Block Ace (DS Pharma, Japan), the serial sections of spleens were incubated with rabbit anti-ATP6V0D2 for 1 h at room temperature and washed with PBS. Horseradish peroxidase (HRP)-conjugated anti-rabbit IgG (Nichirei, Japan) was applied to the sections, incubated for 1 h at room temperature, and washed with PBS. After enzymatic color development was performed using 3,3′-diaminobenzidine (Nichirei) or 4-[(4-amino-m-tolyl) (4-imino-3-methylcyclohexa-2,5-dien-1-ylidene) methyl]-o-toluidine monohydrochloride (new fuchsine, Nichirei), the sections were counterstained with Mayer’s hematoxylin solution for 1 min and rinsed with tap water.

### Giemsa staining

BMDMs cultivated on chamber slides were fixed with methanol and stained with 5% Giemsa solution (Sigma, USA) diluted in distilled water for 20 min. After air drying, the slides were rinsed with xylene and then mounted in Mount Quick (Daido Sangyo, Japan).

### Analysis of MGC formation

BM cells were cultivated on eight-well chamber slides (Thermo Fisher, USA) at a density of 2 × 10^6^ cells/ml. The day 7 BMDMs were stimulated with 20 ng/ml of IL-4, 20 ng/ml of GM-CSF, 100 ng/ml of LPS, or their combinations. In parallel, the BMDMs were infected with CytoRed-labeled *Leishmania* promastigotes at MOI of 20, and extracellular parasites were washed off at 24 h. After 72 h from the initial treatment, the cytokine-stimulated or *Leishmania*-infected BMDMs were fixed by 4% paraformaldehyde in PBS and stained with Hoechst 33342 dye (Dojindo) after incubation. Digital pictures were taken with a fluorescent microscope (BZ-X810: Keyence, Japan) for the quantification of cells and cell nuclei. Cells with two or more nuclei were regarded as MGCs. The images from three independent experiments were evaluated. Over 100 cells were counted in every stimulation group. One-way ANOVA followed by Dunnett’s multiple comparisons test was used for the statistical analysis between the control group and the stimulation groups.

### *In vitro* hemophagocytosis and bead phagocytosis assay

BM cells were cultivated on eight-well chamber slides (Thermo Fisher) at a density of 1 × 10^6^ cells/ml. The day 7 BMDMs were stimulated for 72 h with 15 ng/ml of IL-4, 20 ng/ml of GM-CSF, 20 ng/ml of IFN-γ, and 1,000 ng/ml of LPS or infected with *L. donovani* promastigotes at MOI of 20 and incubated for 72 h as described above. After 72 h of stimulation, the cells were incubated with CytoRed-labeled mouse RBCs or latex beads (Polysciences, USA) for 2 h. The cell nuclei were counterstained with Hoechst 33342, and counting of hemophagocytes or bead-phagocytosing cells was performed using BZ-X810.

### Quantitative RT-PCR

For the quantitative RT-PCR, RNA was extracted following the manufacturer’s instructions using TRIzol reagent (Invitrogen). The concentration of total RNA was measured by DU730 Life Science UV/vis spectrophotometer (Beckman Coulter, USA). Four micrograms of total RNA was used as a template for the synthesis of 20 μl of cDNA. A tube containing 500 ng of oligo (dT)16 and 10 nmol of dNTPs (Fisher Scientific, UK) with template RNA was incubated for 5 min at 65°C at a 13-μl reaction volume. After adding 5× first-strand buffer, 200 nmol of DTT (Thermo), and 200 U of M-MLV (Thermo), the tube was incubated at 37°C for 50 min. The reaction was inactivated by incubation for 15 min at 70°C. The synthesized cDNA was used for the expression analyses of murine *Atp6v0a2*, *Atp6v0b*, *Atp6v0d1*, *Atp6v1a*, *Atp6v0d2*, *Atp6v1d*, *Atp6v1f*, and *β-actin*. The designed primers are listed in **Table S1**. A real-time polymerase chain reaction (PCR) assay was carried out using 1 μl of reverse transcription PCR product as the template and 10 μl of SYBR Select Master Mix (Thermo) on the ABI Prism 7000 Sequence Detection System (Thermo). Data were analyzed by 2−ΔΔCt methods through normalization with murine *β-actin*. The thermal cycling conditions were 94°C for 10 min, followed by 40 cycles at 94°C for 15 s and 60°C for 1 min.

### Western blotting

Day 7 BMDMs (2 × 10^6^) were infected with 4 × 10^7^ cells of *L. donovani* and incubated for 48 h at 37°C. After washing three times with PBS, the macrophages were lysed in SDS sample buffer with 5% dithiothreitol addition, boiled for 5 min and separated by 12% SDS-polyacrylamide gel electrophoresis, and then transferred to a polyvinylidene difluoride membrane (GE Healthcare Bio-Sciences, USA). After blocking with 4% skim milk, the membrane was probed with rabbit anti-ATP6V0D2 antibody (Sigma, 1:2,000 dilution) and rabbit anti-GAPDH antibody (GeneTex, 1:2,500 dilution) diluted with PBS containing 0.05% Tween 20 (PBS-T) plus 10% Block Ace. After washing the membrane with PBS-T three times, it was probed with HRP-linked donkey anti-rabbit IgG antibody (GE Healthcare) at 1:10,000 dilution with PBS-T containing 10% Block Ace. Bands were visualized by an enhanced chemiluminescence detection system (GE Healthcare) and analyzed by LAS-3000 mini (Fujifilm, Japan). Densitometric analysis was performed using ImageJ software from the National Institute of Health.

### Immunofluorescence assay

An immunofluorescence assay was performed to characterize the feature of *L. donovani*-induced MGCs. Macrophages were either unfixed or fixed/permeabilized using Cytofix/Cytoperm fixation and permeabilization solution kit (Becton Dickinson, USA) before staining with antibodies. After blocking with 5% bovine serum albumin (BSA) for 2 h, PE-labeled rat anti-mouse CD11b, PE-labeled rat anti-mouse F4/80, or PE-labeled rat anti-mouse LAMP1 (Biolegend, USA) was applied to the cells and incubated at 4°C for 1 h. For staining with unlabeled antibodies including rat anti-mouse MOMA2 (Abcam, USA), rabbit anti-mouse ATP6V0D2, and rabbit anti-mouse IgG isotype control (Millipore, Germany), the primary antibody was applied to the fixed cells and incubated for 1 h at 4°C followed by the secondary antibody, either Alexa 488-goat anti-rabbit IgG or Alexa 546-goat anti-rabbit IgG (Invitrogen), for 1 h and counterstained with Hoechst 33342.

For double staining of ATP6V0D2 and mitochondria and ER, BMDMs were first incubated with MitoTracker Red and ER-Tracker Red (Invitrogen) and washed before fixation according to the manufacturer’s instructions.

Line graphs of optical sectioning images with multiple fluorescence tunnels were plotted and analyzed using Fiji software. The fluorescence density of the stained cell images was analyzed using Keyence BZ-X800 analyzer software. The red fluorescence tunnel images were used with a cutoff threshold of 5 to cover the area of a single cell for analyzing the light brightness of each separated area.

### Expression constructs and transfection

An expression construct for the murine *Atp6v0d2* gene (GenBank Accession No. NM_175406.3) was prepared in the pCAG expression vector to simultaneously express the enhanced green fluorescence protein (EGFP) gene. We first constructed the pCAG-IRES-GFP-neo plasmid vector by inserting the IRES-GFP (Yoshioka et al., 2015) fragment with *Bam*HI–*Cla*I site at the 5′ side of IRES into the *Eco*RI site of pCAG-T3-hCAS-pA (Addgene# 48625) (Fujii et al., 2013). Then, its *Sal*I–*Hin*dIII fragment encoding CAG-IRES-GFP was inserted into the same restriction sites of pGK-Neo-pA (Addgene #13442) (Soriano et al., 1991). The *Atp6v0d2* gene was PCR-amplified from cDNA from the spleen of an *L. donovani*-infected mouse using the proofreading Platinum SuperFi DNA polymerase (Invitrogen) and the following primers, 5′-TAA GGA TCC ACC ATG CTT GAG ACT GCA GAG CT-3′ (forward) and 5′-TAA ATC GAT TTA TAA AAT TGG AAT GTA GC-3′ (reverse), encompassing the start and stop codons (shown in bold) and containing added *Bam*HI and *Cla*I restriction sites (underlined). The amplification reaction product was cloned into the pCAG-IRES-GFP-neo expression vector using the restriction sites as previously mentioned. For transfection, 0.5 μg of DNA of the expression vector was formulated with 1.5 μl of polyethylenimine (PEI) in 30 μl of DMEM. The mixture was kept at room temperature for 20 min and then applied to BMDMs. After incubating with the PEI–vector mixture for 4 h, the culture supernatant was removed and replaced with a fresh antibiotic-free medium for an additional 48-h cultivation. The transfection efficiency was checked by EGFP fluorescence using BZ-X810.

### RNA interference

RNA interference was performed with the following small interfering RNAs (siRNAs): si-Control (#4390843, Invitrogen) and si-ATP6V0D2 (s109716, #4390771, Invitrogen). Six microliters of 10 μM of siRNA was incubated with 9 μl of Lipofectamine RNAiMAX (Invitrogen) in DMEM for 5 min. Day 6 BMDMs (2 × 10^6^) were transfected with the mixture for 72 h. For the *Leishmania* infection group, the transfected BMDMs were incubated with *L. donovani* 24 h after transfection.

### Statistical analysis

Statistical comparisons were performed by one-way ANOVA followed by Dunnett’s multiple comparison test or unpaired *t*-test with GraphPad Prism 9 software (GraphPad Software, USA). A difference between groups was considered statistically significant when the *P*-value was less than 0.05.

## Results

### *Leishmania* infection promoted multinucleated giant cell formation

**Figure 1:**
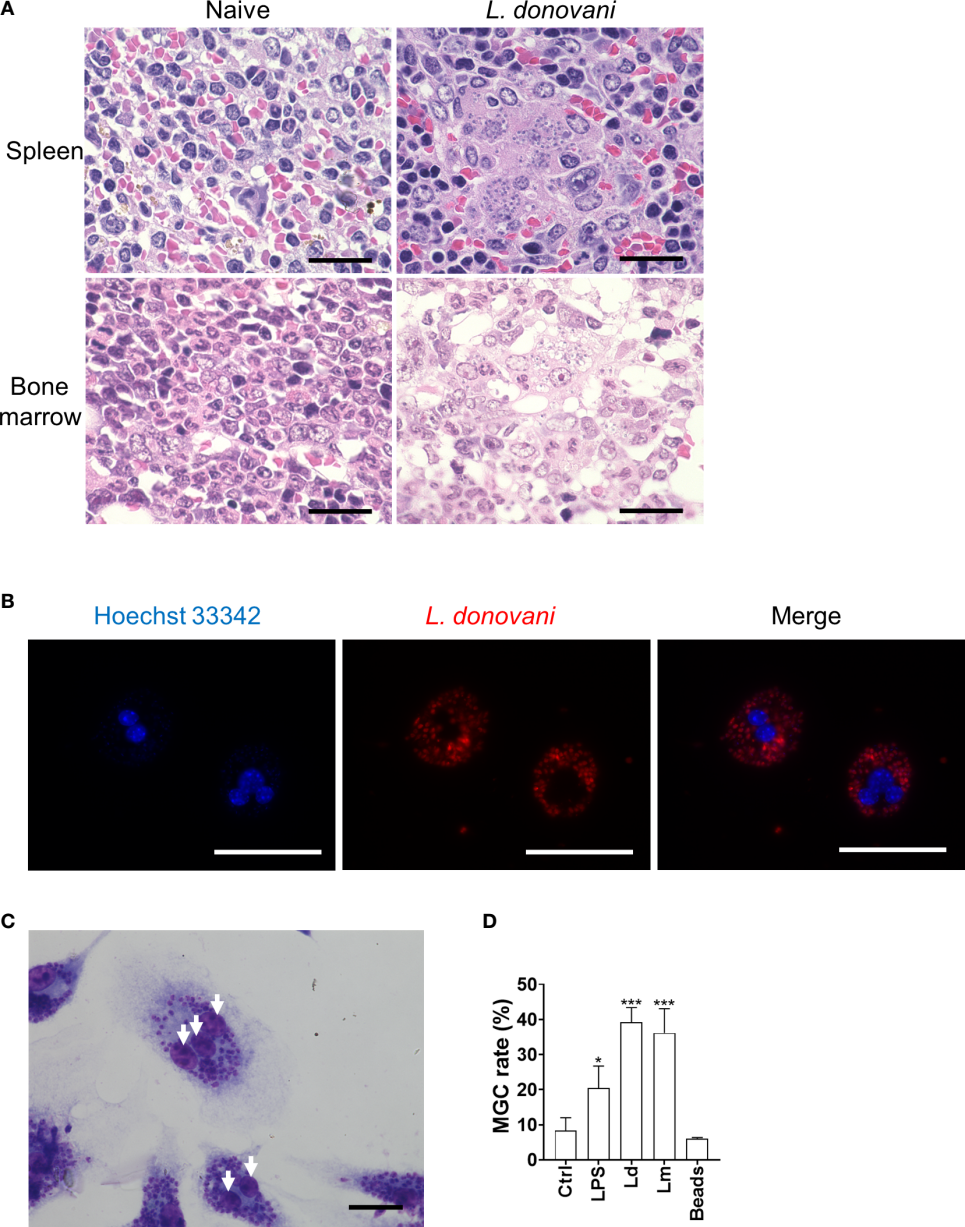
*Leishmania* infection promotes macrophage multinucleation. **(A)** Micrographs of H&E-stained sections of the spleen and bone marrow from naive or *Leishmania donovani*-infected BALB/cA mice at 24 weeks post-infection are shown. Bars, 20 μm. **(B)** Multinucleated giant cells (MGCs) with CytoRed-labeled *L. donovani* amastigotes. Nuclei were stained with Hoechst 33342. Bars, 50 μm. **(C)** Giemsa-stained bone marrow-derived macrophages (BMDMs) infected with *L. donovani*. Bar, 20 μm. White arrows, nuclei. **(D)** BMDMs were either untreated, stimulated with LPS, infected with *L. donovani* or *L. major*, or cultivated with latex beads for 72 h. Cells with two or more nuclei were counted as MGCs. MGC rates were calculated by analyzing over 100 cells in each experiment, and the mean + SD of three independent experiments is shown. *P < 0.05, ***P < 0.001 vs. the control group by one-way ANOVA followed by Dunnett’s multiple comparisons test.

As previously reported, the microscopic observation of the H&E-stained section of the spleen and bone marrow of BALB/c mice at 24 weeks after *L. donovani* infection revealed the formation of amastigote-harboring MGCs that often phagocytose erythrocytes (**Figure 1A**).

To examine whether *Leishmania* infection promotes multinucleated giant cell formation directly, *in-vitro* infection of BMDMs with *L. donovani* and *L. major* was performed, and the MGC formation rate was calculated at 72 h post-infection. MGC was defined as a cell that had two or more nuclei in this research. Compared to BMDMs without treatment, it was observed that more MGCs were formed in the *L. donovani* infection group (**Figures 1B, C**). MGCs accounted for 39.2% ± 4.2% and 36.2% ± 6.9% of the total macrophages in *L. donovani-*infected and *L. major*-infected macrophages, respectively (**Figure 1D**), while the multinucleation rate in macrophages without treatment was 8.3% ± 3.6%, and in macrophages incubated with beads, the rate was 6.0% ± 0.4%.

### MGCs induced by *L. donovani* infection had hemophagocytic activity

**Figure 2:**
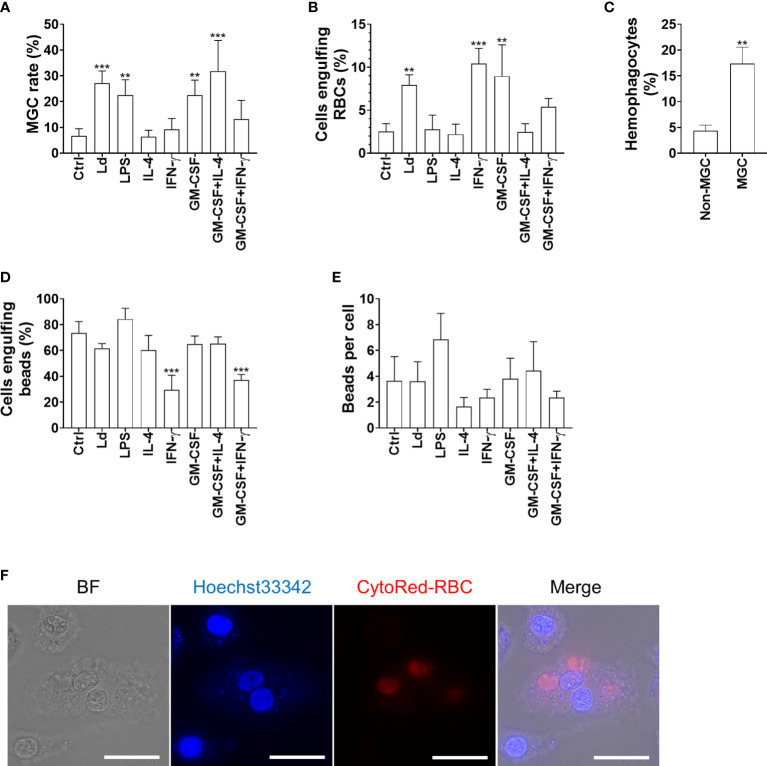
Hemophagocytic characteristics of *Leishmania*-induced MGCs. **(A)** BMDMs were treated as indicated and analyzed for MGC formation. Cells that had two or more nuclei were counted as MGCs. Over a hundred cells of each stimulation group were analyzed, and the mean + SD of three independent experiments is shown. **(B, C)** The BMDMs were incubated with RBCs and examined for hemophagocytosis. **(B)** The proportion of hemophagocytes in each treatment group is shown. **(C)** The proportions of hemophagocytes in non-MGCs and MGCs of *L. donovani*-infected BMDMs are shown. **(D, E)** The BMDMs were incubated with latex beads and examined for phagocytosis. **(D)** The proportion of cells phagocytosing beads in each treatment group is shown. **(E)** The average number of phagocytosed beads in each treatment group is shown. **P < 0.01, ***P < 0.001 vs. the control group by one-way ANOVA followed by Dunnett’s multiple comparisons test. **(F)** MGC engulfing RBCs. The RBCs were labeled with CytoRed. Bars, 20 μm.

To further characterize MGCs that were induced by *Leishmania* infection, we compared their hemophagocytosis and phagocytosis ability with BMDMs treated with cytokines including GM-CSF, IL-4, and IFN-γ, which are commonly known as multinucleation inducers leading to the formation of phenotypically distinct MGCs. While BMDMs cultivated with IL-4 alone, IFN-γ alone, or GM-CSF + IFN-γ did not show apparent MGC formation, GM-CSF alone (22.5% ± 5.8%), GM-CSF + IL-4 (31.8% ± 11.9%), LPS (22.5% ± 6.0%), and *L. donovani* infection (27.2% ± 4.7%) promoted MGC formation compared with unstimulated BMDMs (6.7% ± 2.7%) (**Figure 2A**). When BMDMs infected with *L. donovani* were co-cultured with erythrocytes, the cells had a higher hemophagocytosis rate compared with uninfected BMDMs (**Figures 2B, F**). The hemophagocytosis was more prominent in MGCs than in cells with a single nucleus in the infected BMDMs (17.4% ± 3.2% and 4.3% ± 1.1%, respectively; **Figure 2C**). BMDMs stimulated with GM-CSF alone or IFN-γ alone or those infected with *L. donovani* showed significantly higher hemophagocytosis rates (9.0% ± 3.7%, 10.4% ± 1.8%, and 7.9% ± 1.2%, respectively), whereas phagocytic activities on latex beads did not increase by these treatments (**Figures 2B, D, E**).

**Figure 3:**
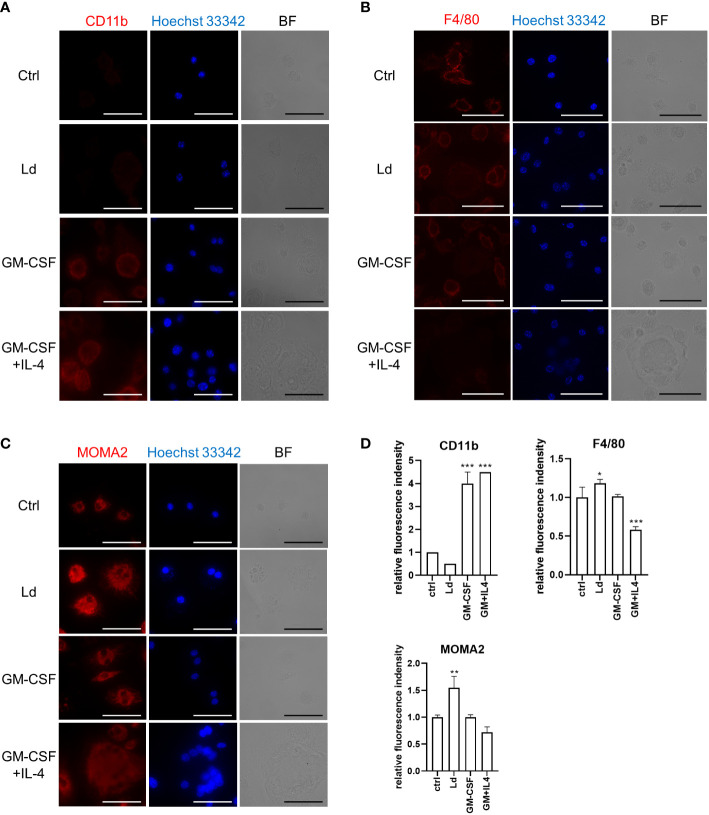
*Leishmania donovani*-induced MGCs have MOMA2^high^CD11b^low^ feature. BMDMs were cultivated on eight-well chamber slides and stimulated with GM-CSF or GM-CSF + IL-4 or infected with *L. donovani*. Cells were fixed/permeabilized at 72 h after stimulation and stained for CD11b. BF, bright field **(A)**, F4/80 **(B)**, and MOMA2 **(C)**. Representative photos of two independent experiments are shown. Bars, 50 μm. **(D)** Fluorescence density of CD11b, F4/80, and MOMA2 per cell. Means + SD of over 20 cells in each group are shown. *P < 0.05, **P < 0.01, ***P < 0.001 vs. the control group by one-way ANOVA followed by Dunnett’s multiple comparisons test.

To further characterize MGCs induced by different stimulations, we performed immunofluorescence staining to compare the expression levels of macrophage markers including CD11b, F4/80, and MOMA2 on these MGCs. *Leishmania donovani*-induced MGCs showed unchanged expression of CD11b compared with the control BMDMs, while the marker molecule was upregulated in MGCs induced by either GM-CSF alone or GM-CSF + IL-4 (**Figure 3A**). F4/80 expression was not apparently different among the control BMDMs or those induced by GM-CSF alone, while it was downregulated in MGCs induced by GM-CSF + IL-4 (**Figure 3B**). A marked increase in MOMA2 expression was found in *L. donovani*-induced MGCs when compared with the other groups (**Figures 3C, D**).

### ATP6V0D2 expression was upregulated in *L. donovani*-infected macrophages

**Figure 4:**
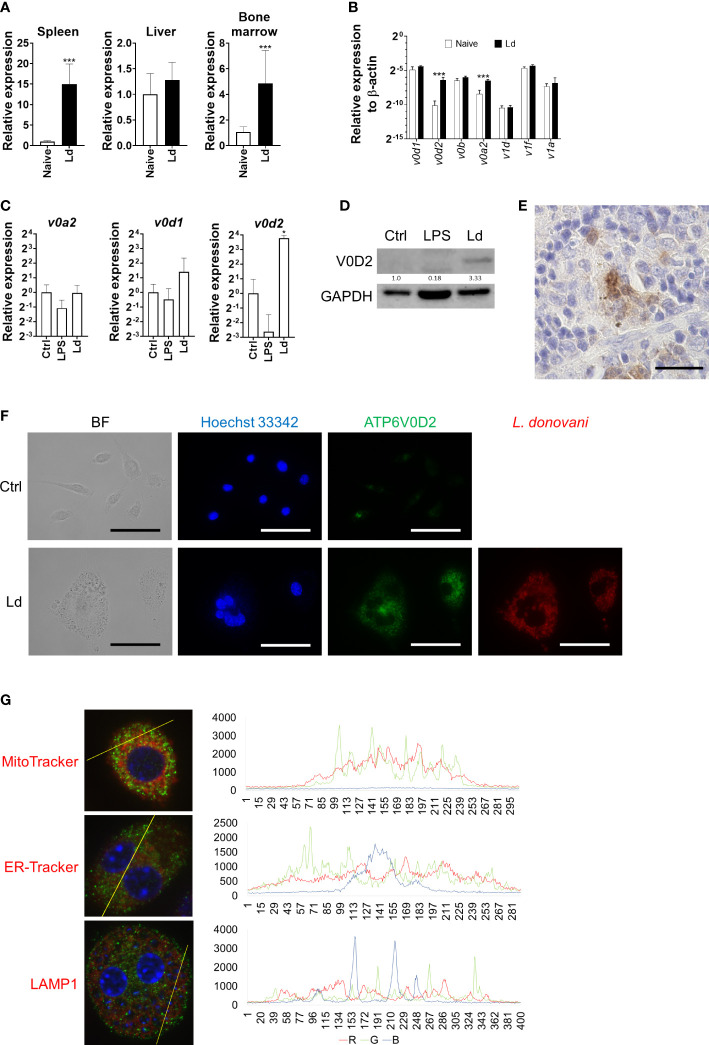
Upregulation of ATP6V0D2 expression by *Leishmania donovani* infection. **(A)** Expression levels of murine *Atp6v0d2* mRNA in the spleen, liver, and bone marrow of naive or *L. donovani*-infected mice were analyzed by qPCR. Means and SD of the representative data from three independent experiments with similar results are shown. ***P < 0.001 by unpaired *t*-test. **(B)** Expression levels of *Atp6v0d1*, *Atp6v0d2*, *Atp6v0b*, *Atp6v1d*, *Atp6v1f*, and *Atp6v1a2* mRNA in the spleen of naive or *L. donovani*-infected mice were quantified by qPCR. Means and SD of the representative data from three independent experiments with similar results are shown. ***P < 0.001 by multiple *t*-tests. **(C)** BMDMs were either stimulated with LPS or infected with *L. donovani* for 48 h, and the expression of *Atp6v0d1*, *Atp6v0d2*, and *Atp6v1a2* mRNA was quantified by qPCR. *P < 0.05 vs. the control group by one-way ANOVA followed by Dunnett’s multiple comparisons test. **(D)** Western blotting for ATP6V0D2 (40 kDa) and GAPDH (35 kDa) in untreated, LPS-stimulated, or *L. donovani*-infected BMDMs. **(E)** ATP6V0D2-positive MGC in the spleen of *L. donovani*-infected mouse, Bars, 50 μm. **(F)** Untreated BMDMs or *L. donovani*-infected BMDMs were stained for ATP6V0D2 with Hoechst 33342 counterstaining. BF, bright field. **(G)** *Leishmania donovani*-infected BMDMs were fixed and stained for ATP6V0D2 coupled with staining with MitoTracker, ER-Tracker, or anti-LAMP1. Representative photos of two independent experiments with similar results are shown. Colocalization analyses were performed using the plot profile tool of ImageJ Fiji. Bars, 50 μm.

To explore the possible factors that are responsible for MGC formation during *Leishmania* infection, the transcriptome of *L. donovani*-infected mice was analyzed, and we found that *Atp6v0d2* was one of the most upregulated genes in the spleen of infected mice (unpublished data). Therefore, the expression of *Atp6v0d2* in the spleen, liver, and bone marrow was further confirmed by quantitative PCR. *Atp6v0d2* expression increased both in the spleen and bone marrow of *L. donovani*-infected mice, 15.0 ± 4.9 and 4.9 ± 2.6 times that in naive mice, respectively, whereas no significant change was observed in the liver (**Figure 4A**). The expression of other V-ATPase components in the spleen of *L. donovani*-infected mice was also measured by qPCR. Among the examined genes, *Atp6v0a2* was the most prominently upregulated gene, and the upregulation was not in parallel with the gene expression of other components most of which were unchanged by the infection (**Figure 4B**).

Upregulation of *Atp6v0d2* expression was also found in BMDMs infected with *L. donovani* (**Figure 4C**). In contrast, BMDMs treated with LPS showed a marked decrease in *Atp6v0d2* expression (**Figure 4C**). ATP6V0D2-positive cells were increased in the spleen of *L. donovani*-infected mice. The expression of ATP6V0D2 was restricted to macrophage-like cells (**Figure 4E**). Induction of ATP6V0D2 expression in BMDMs by *L. donovani* was also confirmed at a protein level by Western blotting and immunofluorescence staining using an anti-ATP6V0D2 antibody (**Figures 4D, F**). Co-staining of ATP6V0D2 and F4/80 on unfixed cells showed that the upregulated ATP6V0D2 did not locate on the cell membrane (data not shown). In fixed and permeabilized cells, ATP6V0D2 was co-stained with MitoTracker, ER-Tracker, and LAMP1 for labeling the mitochondria, endoplasmic reticulum, and lysosomes, respectively, whereas neither of them colocalized with ATPV0D2 (**Figure 4G**).

### Overexpression of ATP6V0D2 promoted MGC formation in BMDMs

**Figure 5:**
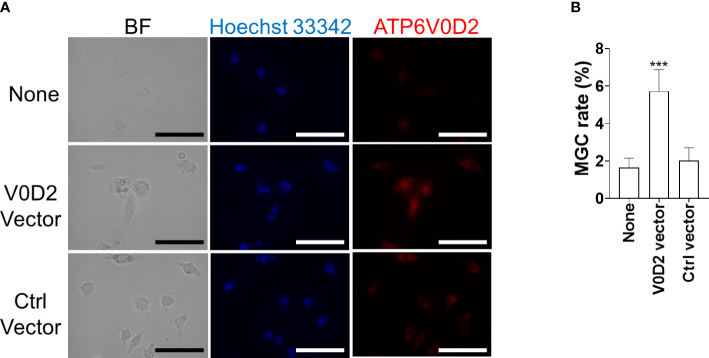
Overexpression of ATP6V0D2 promotes MGC formation in BMDMs. **(A)** BMDMs were transfected with either a vector coding ATP6V0D2 or a control vector and were stained with anti-ATP6V0D2 antibody and Hoechst 33342. BF, bright field. **(B)** Cells with two or more nuclei were counted as MGCs. Over 100 cells for each experiment were counted for calculating MGC rates. Means ± SD of three independent experiments are shown. ***P < 0.001 by one-way ANOVA followed by Dunnett’s multiple comparisons test.

To examine whether multinucleation that was observed in *L. donovani*-infected macrophages is associated with increased ATP6V0D2 expression, we constructed the pCAG-ATP6V0D2 expression vector and transfected it into BMDMs. The upregulated expression of ATP6V0D2 was confirmed in the transfected cells (**Figure 5A**). The MGC formation rate was calculated by counting the proportion of cells that had more than two nuclei. It was significantly higher in ATP6V0D2-overexpressed BMDMs than untreated BMDMs or those transfected with an expression vector coding an irrelevant protein (**Figures 5A, B**).

### Knockdown of ATP6V0D2 suppressed MGC formation in *Leishmania donovani*-infected BMDMs

**Figure 6:**
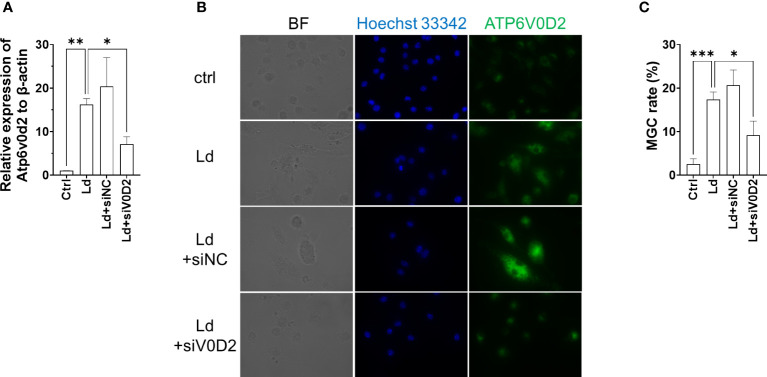
Knockdown of ATP6V0D2 reduced MGC formation in *Leishmania donovani*-infected BMDMs. **(A)** BMDMs were transfected with either siRNA of ATP6V0D2 or random negative control 24 h before *L. donovani* infection. Relative expressions of *Atp6v0d2* mRNA to *β-actin* mRNA were quantified by qPCR. **(B)** ATP6V0D2 was stained with anti-ATP6V0D2 antibody and Hoechst 33342. **(C)** Over 300 cells for each experiment were counted for calculating MGC rates. Means ± SD of three independent experiments are shown. *P < 0.05, **P < 0.01, ***P < 0.001 by one-way ANOVA followed by Dunnett’s multiple comparisons test.

To further address whether ATP6V0D2 is indispensable in the formation of *L. donovani*-induced MGC formation, we knocked down ATP6V0D2 by siRNA in *L. donovani*-infected BMDMs. The suppressed expression of ATP6V0D2 was confirmed in cells transfected with specific siRNA (**Figures 6A, B**). MGC formation in *L. donovani*-infected BMDMs was suppressed when treated with siRNA for ATP6V0D2, while no significant difference was observed in the control siRNA group (**Figure 6C**).

## Discussion

Though it is commonly considered that cytokines are important in deciding the fate of macrophages/monocytes undergoing multinucleation, we observed that infecting macrophages with *Leishmania* was enough for triggering this process. The result that cultivating macrophages with latex beads did not induce multinucleation suggests that MGC formation by *Leishmania* infection is not simply linked to activation of the phagocytosis process in macrophages.

Although the phagocytosis abilities toward latex beads between these MGC phenotypes were similar, *L. donovani*-induced MGCs showed a higher hemophagocytosis rate (**Figure 2**). In immunofluorescence staining, these *Leishmania*-induced MGCs showed a MOMA2^high^CD11b^low^ feature, which was similar to MGCs found in the spleen of *L. donovani*-infected mice (Morimoto et al., 2016) and was different from MGCs induced by GM-CSF alone or GM-CSF + IL-4 (**Figure 3**). Together, it is suggested that *L. donovani* infection induces not only MGC formation but also transformation to pathogenic phagocytes. It is interesting that stimulation with IFN-γ, a known hemophagocytosis inducer in hemophagocytic lymphohistiocytosis with Epstein–Barr virus infection (Jordan et al., 2004), sorely caused enhanced hemophagocytosis rate but no significant change in MGC formation. It indicated that the formation of MGC and hemophagocytosis are not necessarily parallel events, and *L. donovani* is unique to induce both.

MOMA2 expression may represent a unique type of hemophagocytic MGCs that are distinguished from other MGCs. For example, MOMA2 expression is rather downregulated during the differentiation of bone marrow cells to osteoclasts with M-CSF + RANKL (Khapli et al., 2003). MOMA2 is also known as a marker for tingible body macrophages which possess high hemophagocytic activities (Kraal et al., 1987; Gotur and Wadhwan, 2020). In a mouse model of intracerebral hemorrhage, MOMA2-positive MGCs were induced to phagocytose erythrocytes (Wei et al., 2020). Although the target antigen of MOMA2 remains unclear, further characterization of MOMA2-positive macrophages may lead to the elucidation of the boundary between hemophagocytic and non-hemophagocytic MGCs.

To further characterize *Leishmania*-induced MGCs, we focused on ATP6V0D2 in this study because the protein is involved in cell–cell fusion during osteoclastogenesis as well as cytokine-induced MGC formation of macrophages (Lee et al., 2006). We found that the expression of the *Atp6v0d2* gene was markedly increased in the spleen of macrophages of *L. donovani*-infected mice, and the upregulated expression of *Atp6v0d2* was reproduced in BMDMs infected with *L. donovani in vitro* (**Figure 4**). Furthermore, overexpression of ATP6V0D2 in macrophages also promoted multinucleation (**Figure 5**), while knockdown of ATP6V0D2 in *L. donovani*-infected macrophages suppressed MGC formation (**Figure 6**). Moreover, the MOMA2 expression was also upregulated in ATP6V0D2-overexpressed MGCs (**Figure S2**). Together, these results suggested that *Leishmania* infection-induced upregulation of ATP6V0D2 promoted macrophage fusion to form MGCs both *in vitro* and *in vivo*. Interestingly, our results showed that LPS stimulation induced macrophage multinucleation as well (**Figure 1D**). LPS is another multinucleation inducer that increases the expression of RANKL and leads to osteoclast maturation through TLR signaling (Kikuchi et al., 2001). While LPS stimulation also downregulated ATP6V0D2 expression (**Figure 4C**), it suggests that multinucleation can be induced in variant pathways, and the characteristics of multinucleated macrophages are possibly decided by different causative inducers.

ATP6V0D2 is a component of V-ATPase (Pandey et al., 2007). Therefore, we first considered that *Leishmania*-induced ATP6V0D2 was expressed as part of the V-ATPase located in the lysosomes. However, the results obtained in this study were rather contradictory to the interpretation. First, the upregulated expression of *Atp6v0d2* in BMDMs infected with *L. donovani* was not accompanied by the upregulation of the other V-ATPase components (**Figure 4**). This unorchestrated change of *Atp6v0d2* expression among V-ATPase components was also reported in macrophages treated with a TLR3 agonist or osteopontin (Zainol et al., 2019; Dai et al., 2022). Second, the location of upregulated ATP6V0D2 did not overlap with a lysosomal marker LAMP1 (**Figure 4**), as opposed to a previous finding in BMDMs (Xia et al., 2019). It did not locate on the plasma membrane as previously reported in osteoclasts (Okayasu et al., 2012). Instead, a great proportion of ATP6V0D2 had a scattered distribution in the cytosol in our study. It is possible that upregulated ATP6V0D2 in *Leishmania*-infected macrophages is produced irrespective of the proton pump and has a distinct location from that of normally expressed ATP6V0D2. In fact, ATP6V0D2 is dispensable for the acidification of lysosomes in macrophages (Xia et al., 2019), and V-ATPase activity is normal in *Atp6v0d2*-deficient mice (Lee et al., 2006). It was also reported that *Atp6v0d2* deficiency did not influence phagosome acidification or parasite burden in *Leishmania amazonesis*-infected macrophages (Kikuchi et al., 2001). On the other hand, some reports presented the involvement of ATP6V0D2 in endosome acidification (Wu et al., 2009; Murase et al., 2018), suggesting that the role of ATP6V0D2 is variable in different conditions.

It is still unknown how ATP6V0D2 is involved in MGC formation during *Leishmania* infection. At least, the MGC formation found in this study should be beneficial to the parasites since the MGCs induced by *L. donovani* are hemophagocytic (**Figure 2**) and the uptake of erythrocytes by macrophages leads to superior intracellular survival of the parasites (Morimoto et al., 2019). Therefore, elucidation of the mechanisms for *Leishmania*-induced upregulation of ATP6V0D2 may lead to the control of parasite infection. Our finding of *Atp6v0d2* downregulation by LPS is consistent with the reports by other groups (Pessoa et al., 2019; Xia et al., 2019). Although *Atp6v0d2* upregulation is reported on macrophages infected with *L. amazonesis* (Kikuchi et al., 2001), the magnitude of upregulation was less than two-fold and much weaker than that found in *L. donovani* infection *in vitro* and *in vivo*, indicating the involvement of species-specific molecules of the parasites for induction of ATP6V0D2 in macrophages. It is reported that *Atp6v0d2* expression is controlled by TFEB (Palmieri et al., 2011; Liu et al., 2019), NFATc1 (Kim et al., 2008; Oh et al., 2015; Kim et al., 2022), and ELAVL1 (Zainol et al., 2019). In the spleen of *L. donovani*-infected mice, we did not find transcriptional changes in any of Tfeb, Nfatc1, or Elavl1 (data not shown). The results may not be contradictory as the activities of both transcriptional factors are regulated not only by their amount but also by phosphorylation status.

In addition to the upstream of *Atp6v0d2*, elucidation of gene regulation downstream of *Atp6v0d2* is indispensable for understanding the induction of hemophagocytic MGCs by *Leishmania* infection. ATP6V0D2 is involved in inflammasome activation, and its defect results in susceptibility to *Salmonella* infection (Xia et al., 2019). It also functions to limit inflammasome activation in liver ischemia–reperfusion injury (Wang et al., 2021). Moreover, ATP6V0D2 is also reported as a key factor of Toll-like receptor 4 signaling that LPS-induced production of proinflammatory cytokines by macrophages is also limited by ATP6V0D2, but intriguingly, the production of such cytokines in stimulation with TLR3, TLR7, or TLR9 agonists is rather enhanced by ATP6V0D2 (Murase et al., 2018). The regulation of ATP6V0D2 and the resulting outcomes in macrophages seem complicated, and further studies are necessary to understand how the molecule is involved in MGC formation and their acquisition of hemophagocytic characteristics.

## Data availability statement

The original contributions presented in the study are included in the article/**Supplementary Material**. Further inquiries can be directed to the corresponding author.

## Ethics statement

The animal study was reviewed and approved by Animal Experiment Committee at the University of Tokyo.

## Author contributions

Conceptualization, JH and YG; methodology, JH, CS, WF, JY, and YG; investigation, JH, WF, JY, and YG; formal analysis, JH, JY, and YG; resources, CS, WF, and YG; data curation, JH, JY, and YG; writing—original draft preparation, JH; writing—review and editing, YG; supervision, YG; funding acquisition, YG. All authors have read and agreed to the published version of the manuscript.

## Funding

This work was supported by KAKENHI (18H02649, 20K21516, 21H02722, 22H05057 to YG) from the Japan Society for the Promotion of Science, a grant from the Global Health Innovative Technology Fund (G2018-111 to YG), and joint research grants from Hokkaido University International Institute for Zoonosis Control to YG.

## Conflict of interest

The authors declare that the research was conducted in the absence of any commercial or financial relationships that could be construed as a potential conflict of interest.

## Publisher’s note

All claims expressed in this article are solely those of the authors and do not necessarily represent those of their affiliated organizations, or those of the publisher, the editors and the reviewers. Any product that may be evaluated in this article, or claim that may be made by its manufacturer, is not guaranteed or endorsed by the publisher.
