# Supplementary material for: Leishmania infection-induced multinucleated giant cell formation via upregulation of ATP6V0D2 expression
Source: Front Cell Infect Microbiol. 2022 Sep 23;12:953785. doi: 10.3389/fcimb.2022.953785 (PMC9539756; doi:10.3389/fcimb.2022.953785)
Supplement: Supplementary file 1 [file DataSheet_1.pdf]

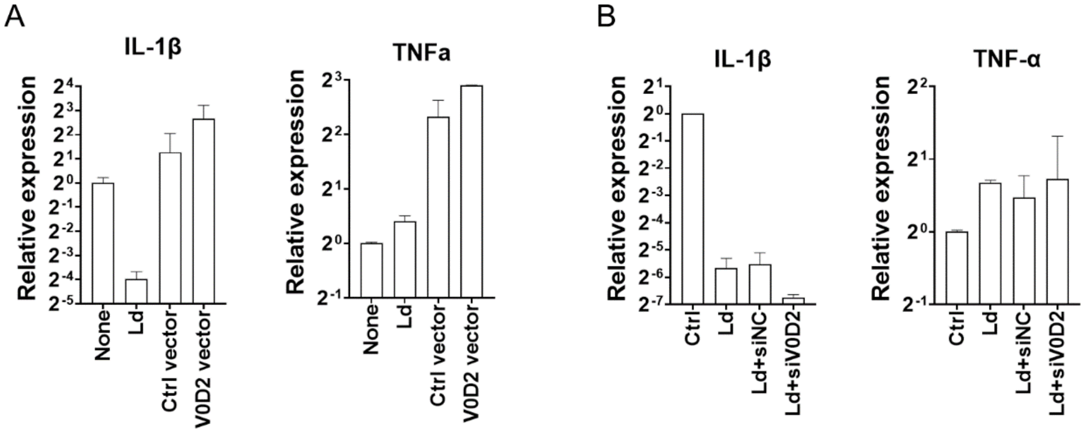

Figure S1. (A) The expression of IL-1β and TNF-α in BMDMs transfected with control vector or ATP6V0D2 vector, or infected with *L. donovani* were measured by qPCR. (B) The expression of IL-1β and TNF-α in BMDMs infected with *L. donovani* plus siRNA treatment were measured by qPCR. siNC, Negative control siRNA; siV0D2, siRNA for ATP6V0D2.

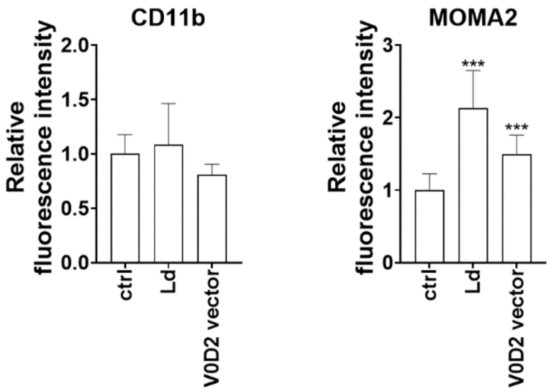

Figure S2. Immunofluorescence intensity of CD11b and MOMA2 in MGCs induced in *L. donovani* infection or ATP6V0D2 overexpression. ***$P < 0.001$ vs. the control group by one-way ANOVA followed by Dunnett's multiple comparisons test

Table S1. Primers used in this study

Primer	Sequence
Atp6v0d2-forward	TGTGTCCCATTCTTGAGTTTGAGG
Atp6v0d2-reverse	AGGGTCTCCCTGTCTTCTTTGCTT
Atp6v0d1-forward	GCTACTTGGAGGGATTAGTGCG
Atp6v0d1-reverse	GCGGAACTCTACTACCATCTTCT
Atp6v0a2-forward	TGGTGCAGTTCCGAGACCT
Atp6v0a2-reverse	GCAGGGGAATATCAGCTCTGG
Atp6v0b-forward	GCATGGTCGTTGTGGGAATCT
Atp6v0b-reverse	GGGAAGTTTCCGTCAGGAACC
Atp6v1a-forward	CCGTACTCCGCACTGGTAAAC
Atp6v1a-reverse	TGGGGATGTAGATACTTTGGGT
Atp6v1d-forward	GGCAAAGACCGGATTGAAATCT
Atp6v1d-reverse	GTCGAAATCGAAGAGTTAAGGCA
Atp6v1f-forward	GCGGGCAGAGGTAAGCTAATC
Atp6v1f-reverse	TTAGGGTGGCGGTTCTTGTTT
βactin-forward	TGGCCATCTCCTGCTCGAA
βactin-reverse	GTTACCAACTGGGAGGACA
IL-1β-forward	GAAAGACGGCACACCCACCCT
IL-1β-reverse	GCTCTGCTTGTGAGGTGCTGATG
TNF-α-forward	GCCTCTTCTCATTCCTGCTTG
TNF-α-reverse	GGGTCTGGGCCATAGAACTG
